# Emotion Dysregulation and Conspiracy Beliefs about COVID-19: The Moderating Role of Critical Social Media Use

**DOI:** 10.3390/ejihpe12100109

**Published:** 2022-10-20

**Authors:** Cristiano Scandurra, Rosa Pizzo, Luca Emanuel Pinto, Claudia Cafasso, Renata Pellegrini, Federica Cafaggi, Oriana D’Anna, Benedetta Muzii, Vincenzo Bochicchio, Nelson Mauro Maldonato

**Affiliations:** 1 Department of Neurosciences, Reproductive Sciences and Dentistry, University of Naples Federico II, 80131 Napoli, Italy; 2 Intradepartmental Program of Clinical Psychology Federico II, University Hospital, 80131 Napoli, Italy; 3 Department of Humanistic Studies, University of Naples Federico II, 80133 Napoli, Italy; 4 Department of Humanistic Studies, University of Calabria, 87036 Rende, Italy

**Keywords:** COVID-19, emotion regulation, conspiracy theories, social media, infodemic, media literacy

## Abstract

As COVID-19 has spread worldwide, conspiracy theories have proliferated rapidly on social media platforms, adversely affecting public health. For this reason, media literacy interventions have been highly recommended, although the impact of critical social media use on the development of COVID-19 conspiracy theories has not yet been empirically studied. Moreover, emotional dysregulation may play another crucial role in the development of such theories, as they are often associated with stress, anxiety, lack of control, and other negative emotions. Therefore, the aim of this study was to test the hypothesis that emotion dysregulation would be positively associated with conspiracy beliefs about COVID-19 and that critical use of social media would attenuate this association. Data from 930 Italian participants (339 men and 591 women) were collected online during the third wave of the COVID-19 outbreak. A moderated model was tested using the PROCESS Macro for SPSS. Results showed that: (1) emotion dysregulation and critical social media use accounted for a significant proportion of the variance in conspiracy beliefs about COVID-19; and (2) critical social media use moderated the effect of emotion dysregulation on conspiracy beliefs about COVID-19. Implications for preventing the spread of conspiracy theories are discussed.

## 1. Introduction

With the global spread of the Coronavirus Disease 2019 (COVID-19), conspiracy theories have spread rapidly on social media platforms, adversely affecting public health [1]. Conspiracy theories can be defined as attempts to explain significant social and political events in terms of secret plots of powerful and malevolent entities acting together against the collective good [2]. Conspiracy beliefs are prevalent in times of crisis, when people seek ways to cope with uncertainty and make sense of ambiguous and unpredictable events [3]. Since the COVID-19 outbreak dramatically increased anxiety and feelings of worry and uncertainty [4,5,6,7,8], it created the “ideal” conditions for the development of conspiracy theories [9]. In such a scenario, where uncertainty is high and the information system is contradictory and often ambiguous, people may turn to alternative explanations to regain control over their environment and restore a sense of predictability [9,10].

Based on the extensive literature suggesting that negative affective states play an important role in the formation of conspiracy theories [2,11,12], the current study aimed to examine the extent to which interindividual differences in emotion regulation contribute to conspiracy belief. In addition, because conspiracy theories are mainly spread through online social networks, interventions to promote social media literacy have been strongly recommended [1,13]. To date, however, there has been no empirical investigation of the impact of critical social media use on the development of conspiracy beliefs related to the COVID-19 pandemic. Therefore, this study attempts to fill this gap in the literature by also examining the role of critical social media use as a protective factor against the development of such theories. 

### 1.1. COVID-19 Conspiracy Theories and the Role of Social Media Consumption

Immediately after the first news about COVID-19, conspiracy theories emerged on social media, becoming more widespread and persistent [1]. The most popular COVID-19 conspiracy beliefs concern the origin of SARS-CoV-2 and assume that the virus was artificially created in a laboratory as a government biological weapon [14] or that the virus was intentionally spread by the largest pharmaceutical companies for profit [10]. There are also numerous narratives circulating on social media suggesting that governments are hiding the truth about the pandemic and using the emergency to pursue their own interests [15], or that the pandemic is a hoax and the preventive measures imposed by governments are designed to achieve political goals [16].

Recent research has shown that conspiracy beliefs about COVID-19 have detrimental consequences for public health [17,18]. Specifically, COVID-19 conspiracy theories were found to be negatively associated with recommended health behaviors (i.e., social distancing and wearing a face mask) and with intentions to undergo diagnostic or antibody testing and vaccination [19,20,21,22]. Because conspiracy theories can have a negative impact on people’s willingness to comply with government recommendations, there are concerns about their proliferation on social media [23]. For example, the overwhelming amount of unreliable information about COVID-19 disease has become a serious threat to public health, prompting the World Health Organization [24] to call on governments to take action against this “infodemic.” An infodemic is an overabundance of information—some accurate, some unverified and questionable—that can lead to confusion and fatigue, making it difficult for individuals to find trustworthy sources and distinguish accurate information from false and low-quality content [24,25]. In the absence traditional editorial control mechanisms to ensure appropriate content quality, social media has been identified as a major potential vector for the spread of misinformation [26]. 

In this context, the frequency of social media use as a source of information about the COVID-19 pandemic has been positively associated with greater support for COVID-19 conspiracy theories [23,27,28], but little is known about the specific pathways linking social media news consumption to conspiracy beliefs. As previous research has shown, different types of information could influence media users’ susceptibility to conspiracy theories [29]. A crucial role could be played by media literacy, which is a combination of a person’s knowledge about media, motivations, and needs that arise from consuming new media, and intellectual skills needed to critically analyze and interpret information [30,31]. There is evidence that higher media literacy is associated with lower advocacy of conspiracy theories [32,33,34,35], but the limited existing data focus on a general definition of media literacy and leave out the issue of media literacy in the specific context of social media networks. 

As social media platforms have been identified as breeding grounds for conspiracy theories, scholars have emphasized the need to teach social media literacy, and a number of interventions have been developed to empower people to reject fake news and consume and share high-quality information [1,13,36]. Particular attention has been paid to critical media consumption as one of the core components of media literacy, which specifically refers to media consumers’ ability to question the credibility of social media content and to synthesize information from different media channels [37]. There is evidence of the relationship between conspiracy beliefs and constructs closely related to critical social media skills (e.g., media skepticism) [29,38,39]. However, the specific role of critical social media consumption in protecting against the development and maintenance of COVID-19 conspiracy beliefs has not yet been empirically investigated.

### 1.2. Emotion Dysregulation and Conspiracy Beliefs

Following the model proposed by Gratz and Roomer [40], emotion regulation can be conceptualized as a multidimensional construct that includes: (a) awareness, understanding, and acceptance of emotions; (b) the ability to control impulsive behaviors when in distress; (c) the ability to use situationally appropriate strategies to modulate the intensity and duration of emotion in the service of desired goals and situational demands, rather than eliminating or avoiding emotions; and (d) the willingness to experience negative affective states in order to pursue meaningful life activities. Deficits in one or more of these dimensions are understood to indicate difficulties in emotion regulation. 

Some authors have speculated that the adoption of conspiracy theories can be viewed as a means of relief or escape from negative emotions. For example, Douglas and colleagues [3] argued that people are drawn to conspiracy theories when important psychological needs are frustrated. According to this theoretical work, conspiracist ideation is driven by epistemic motives—i.e., the need to understand what is happening around us and to find safe and consistent explanations—as well as existential needs for control, security, and making sense of the world when we face threatening situations. 

Along these lines, it has been argued that conspiracy theories can be characterized as deeply emotional theories because they are based on negative emotional experiences rather than rational considerations [41]. Consistent with this view, extensive research has shown that conspiracy beliefs are associated with perceived loss of control, stress, anxiety, and threat perceptions [11,42,43,44,45,46]. Experimental studies also support this view [42,47,48]. Although belief in conspiracies is often conceptualized as an attempt to manage negative emotional experiences, available evidence suggests that this method is not effective, as such theories ultimately fail to provide effective relief of aversive emotions and may even foster a negative feedback loop that leads to a decreased sense of autonomy and control, as well as to heightened levels of anxiety, powerlessness, and existential threat [49,50].

Research conducted in the context of the COVID-19 pandemic appears to confirm previous findings on the relationship between negative emotions and belief in conspiracy theories. Specifically, COVID-19 conspiracy beliefs were found to be positively related to greater anxiety and stronger feelings of powerlessness [18,51]. Furthermore, Jutzi and colleagues [52] showed that manipulating threat perceptions of the COVID-19 pandemic led to higher anxiety, which in turn led to greater endorsement of COVID-related conspiracy theories. In a study by Heiss and colleagues [43], higher threat perception was associated with stronger endorsement of conspiracy theories one month later.

While it is known that conspiracy beliefs are associated with stress, anxiety, fear, and other negative emotions, no study to date has examined the role of individual differences in the regulation of such emotions. It is conceivable that the effects attributed to negative emotions on conspiracy theories are due to differences in strategies for responding to and regulating affective experiences. Along these lines, emotion dysregulation has typically been associated with increased reactivity to stressful situations [53,54], and consequently, numerous recent studies have found that individuals who lack adaptive emotion regulation skills were particularly affected by the COVID-19 crisis [55,56,57,58]. It is therefore likely that individuals with high emotion dysregulation are particularly vulnerable to conspiracy theories in a climate of heightened negative affect because they have difficulty processing negative emotional experiences effectively.

### 1.3. The Current Study

**Figure 1:**
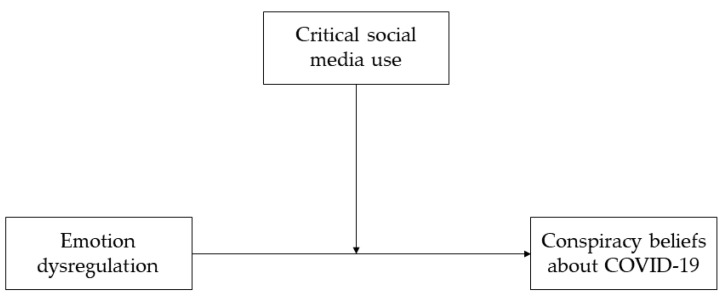
The hypothesized moderation model.

Based on this conceptual and empirical framework, the current study aimed to investigate the role of individual differences in emotion regulation and critical social media use in explaining COVID-19 conspiracy theories in a sample of Italian individuals. To this end, based on the previously highlighted relationship between negative emotions and conspiracy beliefs, we first hypothesized that emotion dysregulation would be positively associated with conspiracy beliefs about COVID-19 (Hypothesis 1). Furthermore, given the well-known role of social media in the spread of conspiracy theories, we expected that critical use of social media would reduce the impact of emotion dysregulation on conspiracy beliefs about COVID-19, and thus moderate this relationship (Hypothesis 2). The hypothesized moderation model is depicted in Figure 1.

## 2. Materials and Methods

### 2.1. Procedures

A quantitative cross-sectional web-based survey was conducted between 27 February and 29 March 2021 during the peak of the third wave of the COVID-19 outbreak in Italy. The survey was posted on the main social media platforms (i.e., Facebook, Telegram, Instagram) and participants were recruited through a snowball sampling recruitment procedure by asking them to forward the link to others. The survey was posted on public pages, in public groups, and in closed groups where opinions and causal beliefs about COVID-19 were explicitly shared. The dissemination of the survey attempted to cover all Italian regions by distributing the invitation link for the questionnaire to online public regional groups with a large number of members.

Participants were informed about the objectives, benefits, and risks of the study, as well as about the researchers. On the first page of the survey, the informed consent form was uploaded, and participants were required to give their consent to participate in the study. To avoid missing data, all questions were mandatory, but participants were free to leave the survey if they wished.

The study was approved by the ethical committee of the University of Naples Federico II (protocol number 9/2021), designed in accordance with the Declaration of Helsinki, and developed in compliance with the EU General Data Protection Regulation.

### 2.2. Participants

Participants were eligible to take the survey if they: (1) were at least 18 years old; (2) had lived in Italy for at least 10 years; and (3) spoke Italian language. A total of 958 participants answered the questions. Of these, 28 did not meet at least one of the first two inclusion criteria. Thus, the final sample consisted of 930 Italian participants. Overall, 339 of the participants were men and 591 were women. The age of the participants ranged from 18 to 78 years (mean (M) = 36.36, standard deviation (SD) = 12.60), and 60.4% (*n* = 562) had a high level of education (≥college degree).

### 2.3. Measures

#### 2.3.1. Emotion Dysregulation

The Difficulties in Emotion Regulation Scale-18 [59] is an 18-item questionnaire assessing emotion dysregulation. Response options ranged from 1 (“almost never”) to 5 (“almost always”), with higher scores indicating greater difficulty in emotion regulation. The scale assesses six dimensions related to emotion regulation (i.e., goals, non-acceptance, impulse, clarity, awareness, and strategies). An example item is “I am confused about how I feel.” For simplicity, we used only the total score in the present study, whose alpha coefficient was 0.89.

#### 2.3.2. Critical Social Media Use 

The 8-item “Critical consuming” subscale of the Social Media Literacy Scale [60] was used to assess critical use of social media. Response options ranged from 1 (“strongly disagree”) to 4 (“strongly agree”), with higher scores indicating a more critical social media use. An example item is “I define information as fake news after reading from various sources.” The alpha coefficient in the current study was 0.82.

#### 2.3.3. Conspiracy Beliefs about COVID-19

The 9-item “Conspiracy Belief Scale” subscale of the COVID-19 Causal Belief Questionnaire [61] was used to assess conspiracy belief about COVID-19. Response options ranged from 0 (“never”) to 10 (“always”), with higher scores indicating stronger conspiracy beliefs about COVID-19. An example item is “The coronavirus is a means to distract citizens from something that is more important.” The alpha coefficient in the current study was 0.91.

### 2.4. Statistical Analyses

Statistical analyses were performed using IBM SPSS Statistics (Version 26), with the significance level set at 0.05. 

First, correlations between variables were calculated using the Pearson’s coefficient. 

Second, a hierarchical multiple regression analysis was performed to test the hypothesis that conspiracy beliefs about COVID-19 are a function of emotion dysregulation, and specifically that critical use of social media would moderate the relationship between emotion dysregulation and conspiracy beliefs. In the first step, emotion dysregulation and critical use of social media were included as independent variables. Then, an interaction term between emotion dysregulation and critical social media use was created by centering both variables and including the interaction in step 2 of the regression model to test whether this interaction added explained variance to the model. 

Third, to assess the conditional effect of emotion dysregulation on conspiracy beliefs about COVID-19 at different levels of critical social media use (−1 *SD*, *M*, +1 *SD*), the PROCESS Macro for SPSS was used, and Model 1 was applied with 10,000 bias-corrected bootstrap samples [62]. This analysis was controlled for age, gender, and education level.

## 3. Results

### 3.1. Descriptive Statistics and Bivariate Correlations

Means, standard deviations, and bivariate correlations between emotional dysregulation, critical social media use, and conspiracy beliefs on COVID-19 are shown in Table 1. The results showed a negative correlation between emotion dysregulation and critical use of social media and between critical use of social media and conspiracy beliefs about COVID-19. In contrast, the results showed a positive correlation between emotion dysregulation and conspiracy beliefs about COVID-19.

### 3.2. Associations between Emotion Dysregulation, Critical Social Media Use, and Conspiracy Beliefs about COVID-19

Hierarchical multiple regression analysis showed that emotion dysregulation and critical social media use accounted for 25% of the variance in conspiracy beliefs about COVID-19 (*R*^2^ = 0.25, *F* (2, 927) = 160.12, *p* < 0.001). Specifically, emotional dysregulation increased the likelihood of believing in a conspiracy about COVID-19 (*b* = 0.47, *p* < 0.001), whereas critical social media use decreased this likelihood (*b* = −0.11, *p* < 0.001). Inclusion of the interaction term between emotion dysregulation and critical social media use slightly increased the explained variance of conspiracy beliefs about COVID-19 (Δ*R*^2^ = 0.02, Δ*F* (1, 926) = 29.05, *p* < 0.001; *b* = −0.16, *p* < 0.001).

### 3.3. Conditional Effect of Emotion Dysregulation on Conspiracy Beliefs about COVID-19 at Different Levels of Critical Social Media Use

**Figure 2:**
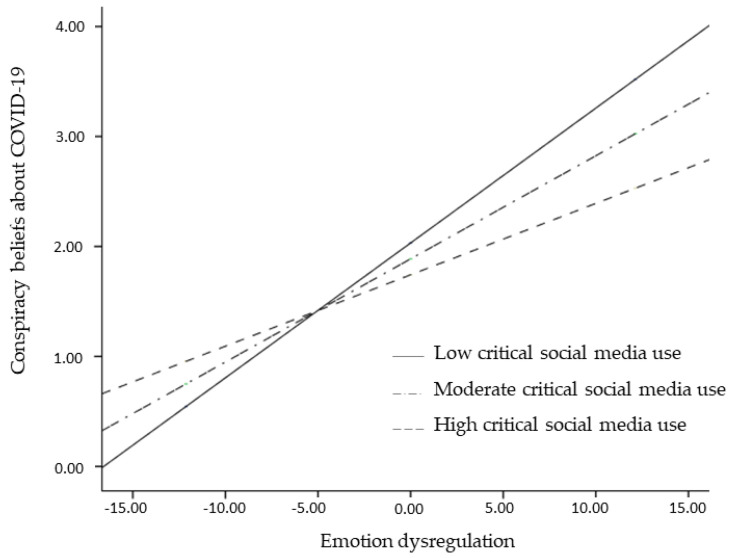
Interaction effect of emotion dysregulation by critical social media use on conspiracy beliefs about COVID-19.

Examination of the interaction plot showed that the effect of emotion dysregulation on conspiracy beliefs about COVID-19 was significant for low (*b* = 0.13, 95% confidence intervals (CI) 0.11, 0.14, *p* < 0.001), moderate (*b* = 0.09, 95% CI 0.08, 0.11, *p* < 0.001), and high (*b* = 0.06, 95% C.I. 0.05, 0.08, *p* < 0.001) levels of critical social media use (Figure 2). This result indicated that critical use of social media may protect individuals with emotion regulation difficulties from the likelihood of developing conspiracy beliefs about the COVID-19. However, this result was particularly true for participants with lower levels of education (*b* = −0.69, *p* < 0.001).

## 4. Discussion

The aim of the present work was to investigate the role of emotion regulation difficulties and social media use in explaining conspiracy beliefs about COVID-19 in a large sample of Italian individuals. To the best of our knowledge, this is the first study to examine the influence of emotional dysregulation on endorsement of conspiracy beliefs and the role of critical social media consumption in moderating this relationship.

In support of our first hypothesis, we found that participants with emotion regulation difficulties were more likely to report conspiracy beliefs. This finding supports the theory that conspiracy beliefs help control acute stress and ward off negative emotions by providing a sense of order, structure, and predictability [2,63]. Our findings are also consistent with empirical studies showing that psychological stress, anxiety, and other negative emotions play a role in predicting endorsement of conspiracy theories [46,64], even during the current COVID-19 pandemic [43,45,51,52]. According to an extensive literature, fearful situations and threatening events can trigger meaning-making mechanisms that focus on reducing unpleasant emotional experiences and regaining a compensatory sense of control, which has been shown to promote conspiracy theory ideologies [2,63,65]. Specifically, elevated levels of anxiety and psychological distress have been shown to make people more likely to engage in heuristics and cognitive patterns that promote conspiracy thinking, such as perceiving illusory correlations beyond unrelated stimuli and making dispositional inferences about others [12,47,48]. Nevertheless, anxiety appears to be associated with heightened threat awareness and a greater propensity to interpret ambiguous information and circumstances in a threatening manner [66], which in turn promotes belief in conspiracy theories [67]. While previous work has shown a significant relationship between negative emotions and conspiracy theories [45,46], our findings are among the first to show a relationship between conspiracy beliefs and difficulties in emotion regulation. If higher levels of stress and anxiety are inevitable when faced with a global crisis such as the COVID-19 pandemic, the present findings suggest that interindividual differences in the experience of and response to stress may be critical in predicting endorsement of conspiracy theories. Indeed, the ability to flexibly regulate emotions is thought to play an important role in stress management [68]. That is, people who rely on dysfunctional emotion regulation processes to cope with situational challenges are more sensitive to threatening and stressful situations [68,69,70].

Recently, difficulties in emotion regulation were also found to be positively associated with increased anxiety and higher levels of psychological distress during COVID-19 outbreak [71,72,73]. Thus, because of difficulty responding adaptively to emotions, individuals with emotion dysregulation may be particularly at risk for developing faulty cognitive thinking patterns in the context of the current COVID-19 pandemic. This is in line with a consistent literature on judgment and decision making, which suggests that individuals with emotion dysregulation are more likely to use biased information processing when faced with stressful situations, with maladaptive emotion regulation processes being positively related to the need to minimize cognitive effort and avoid risk and uncertainty [40,74,75,76]. Accordingly, empirical evidence suggests that intolerance of uncertainty and the need for cognitive closure (i.e., the need to eliminate ambiguity and obtain an answer quickly) promote conspiracy beliefs in situations where clear explanations are lacking [43,77,78]. This could be due to the explanatory power of these theories, which seem to offer ready and comprehensive solutions that promise to resolve ambiguous events through the construction of patterns and meanings. In this sense, conspiracy theories about COVID-19 might be particularly attractive to people with emotion regulation problems, who may turn to such narratives to manage their anxiety and cope with the other negative emotions triggered by the pandemic. In summary, our findings provide preliminary evidence that difficulties in emotion regulation may contribute in part to the acceptance of conspiratorial explanations. Moreover, our results are innovative in finding a novel predictor of conspiracy belief that could have important implications for interventions.

Regarding our second hypothesis, our results showed the moderating effect of critical social media use on the relationship between emotion dysregulation and conspiracy beliefs. Two main considerations can be made in relation to this finding.

First, frequent use of social media has long been associated with higher risk of anxiety and stress symptoms, and recent evidence suggests a positive association between social media use and greater anxiety during the COVID-19 outbreak [79,80]. In particular, it has been suggested that emotion dysregulation may trigger a greater need for information about the pandemic to make the threat more manageable and predictable, with several studies linking difficulties in emotion regulation to increased online search behavior [81,82,83]. However, relying on social media platforms to obtain data about COVID-19 appears to put users at risk of being overwhelmed by an excessive amount of conflicting and often unverified information, which can lead to confusion and reinforce widespread fears and anxieties [84]; as a result, this can trigger a vicious cycle of anxiety in individuals with emotion regulation difficulties. In addition, fake news and conspiratorial articles often aim to fuel shock, anger, and worry by foregrounding sensationalist and emotionally arousing information [85]. This seems to be a crucial factor in explaining the widespread success of conspiracy theories and their great influence on people’s perceptions and attitudes. Previous research has shown that experiencing more emotion can lead to an increased tendency to believe in fake news, and that people who rely on emotion rather than reason are more likely to believe in false headlines [86].

Second, our findings are also consistent with previous studies linking unregulated social media use with endorsement of conspiracy theories and the strength of such beliefs [23,34,35]. These data are not surprising given that conspiracy theories, like most misinformation, are consumed and reinforced primarily through the most popular social media platforms. For example, Allington and colleagues [23] found a positive association between COVID-19 conspiracy beliefs and the extent to which people rely on social media as a source of information about COVID-19. Similarly, numerous recent studies have shown that conspiracy beliefs are associated with an avoidance of traditional and mainstream media sources [87,88]. However, although social media use is widely and consistently recognized as a major risk factor for advocating and spreading conspiracy theories, this does not mean that social media platforms are inherently harmful [89]. Indeed, it may not be the extent of social media use that matters, but rather the quality of the consumer’s information gathering. In this sense, as the recent literature suggests, passive information consumption may play a crucial role in influencing conspiracy theories, while conversely, greater skepticism and lower levels of blind trust in social media news have been shown to be significant moderators of the relationship between social media news use and conspiracy beliefs [29,39]. In this sense, our findings seem to support theoretical work and intervention models that assume that critical consumption of social media can protect against the development of conspiracy beliefs. Our results are also consistent with some previous research conducted outside of the current COVID-19 pandemic that has shown that the higher the level of media literacy, the lower the likelihood of endorsing conspiracy theories [33]. Our study extends these results to specific COVID-19 conspiracy beliefs. While previous research has demonstrated the protective role of general media literacy in the development and maintenance of conspiracy theories [32,33,34,35], our findings represent a new contribution to the literature because here we considered critical media literacy in the specific context of social media.

Regarding sociodemographic data, our results showed that agreement with coronavirus-specific conspiracy beliefs varied according to educational level, which is consistent with previous studies [90,91]. It is well documented that lower levels of education are associated with lower levels of analytical thinking skills, greater feelings of powerlessness, and greater feelings of disadvantage and distrust of institutions [92]. These are factors known to increase people’s susceptibility to believing conspiracy claims [92]. Our findings indicate that lower educational attainment is a risk factor that increases susceptibility to conspiracy belief and, as such, could be considered when planning intervention programs.

Our findings should be read in light of several limitations. First, this was a cross-sectional study, which does not allow for conclusive inferences about the temporality and causality of relationships among variables. Second, participants were recruited during a specific phase of the COVID-19 outbreak (i.e., the third wave). Both limitations should prompt researchers to conduct longitudinal studies to identify cause-and-effect relationships between variables and to assess the validity of the present model during different waves. In addition, participants were recruited through Facebook and other social media platforms, so, although the sample was large, it cannot be considered representative of the Italian population. Social media websites are often used as a sampling tool, and since they are an important means of spreading misinformation, they can easily reach groups of people inclined to conspiracy theories [93]. Notwithstanding, this sampling strategy limited our results to individuals who were active on social media. Future research should replicate our study with more representative samples of the general population. Finally, critical social media use was measured via a self-report questionnaire that may not reflect participants’ actual abilities to critically consume social media content. Future research would therefore benefit from multi-method designs that combine self-report with other measures to test both perceived and actual abilities.

## 5. Conclusions

Despite its limitations, this study adds to the growing literature on the antecedents of conspiracy belief by demonstrating the role of emotion dysregulation and critical use of social media. This confirms the usefulness of intervention strategies aimed at promoting healthy critical behavior (e.g., actively examining information and seeking out credible sources of information) in response to the current infodemic. However, social media literacy programs focus mainly on vigilance, i.e., the cognitive ability to critically analyze news, and ignore the crucial role of emotions in decision making [94,95], which may explain why such interventions have had limited success in countering conspiracy theories. Our findings highlight the urgent need for a multimodal approach that not only targets cognitive factors but can also improve emotion regulation. Helping people develop adaptive coping with negative emotions may prove promising in stopping the spread of conspiracy theories.

## Figures and Tables

**Table 1:** Correlations between emotion dysregulation, critical social media use, and conspiracy beliefs about COVID-19.

	1	2	3	M	SD
1. Emotion dysregulation	1			40.13	12.13
2. Critical social media use	−0.21 ***	1		2.95	0.68
3. Conspiracy beliefs	0.49 ***	−0.21 ***	1	1.96	2.57

Notes: M = Mean; SD = Standard Deviation. *** *p* < 0.001.

## Data Availability

Data will be made available upon reasonable request to the corresponding author.
